# Integrated Raman Laser: A Review of the Last Two Decades

**DOI:** 10.3390/mi11030330

**Published:** 2020-03-23

**Authors:** Maria Antonietta Ferrara, Luigi Sirleto

**Affiliations:** National Research Council (CNR), Institute of Applied Sciences and Intelligent Systems, Via Pietro Castellino 111, 80131 Naples, Italy; antonella.ferrara@na.imm.cnr.it

**Keywords:** nonlinear optics, stimulated Raman scattering, lasers, microphotonics, nanophotonics, nonlinear waveguide, optical microcavity, photonics crystals, nanocrystals

## Abstract

Important accomplishments concerning an integrated laser source based on stimulated Raman scattering (SRS) have been achieved in the last two decades in the fields of photonics, microphotonics and nanophotonics. In 2005, the first integrated silicon laser based upon SRS was realized in the nonlinear waveguide. This breakthrough promoted an intense research activity addressed to the realization of integrated Raman sources in photonics microstructures, like microcavities and photonics crystals. In 2012, a giant Raman gain in silicon nanocrystals was measured for the first time. Starting from this impressive result, some promising devices have recently been realized combining nanocrystals and microphotonics structures. Of course, the development of integrated Raman sources has been influenced by the trend of photonics towards the nano-world, which started from the nonlinear waveguide, going through microphotonics structures, and finally coming to nanophotonics. Therefore, in this review, the challenges, achievements and perspectives of an integrated laser source based on SRS in the last two decades are reviewed, side by side with the trend towards nanophotonics. The reported results point out promising perspectives for integrated micro- and/or nano-Raman lasers.

## 1. Introduction

Nonlinear optical devices enable the control of light by light. Since photons do not interact directly, interaction is possible only by taking advantage of a suitable nonlinear optical material. This is the key element in any nonlinear process, governing the type of nonlinear phenomena supported, the efficiency, size speed, and power characteristics [1,2,3].

In nonlinear optical devices, third order nonlinear effects play a fundamental role. They are due to an induced material polarization, which is proportional to the third power of the electric field, and they can be divided in two class [1,2,3,4]. The first one is related to the Kerr-effect, i.e., the intensity dependence of the refractive index of the medium, occurring in three effects: Self-Phase Modulation (SPM), Cross-Phase Modulation (CPM) and Four-Wave Mixing (FWM). The second one is due to inelastic-scattering phenomenon, which can induce a stimulated effect such as Stimulated Raman-Scattering (SRS). SRS, observed in 1962 for the first time [5,6,7], is given by the interaction between the guided wave and high-frequency optical phonons. SRS depends on the pump intensity and on a gain coefficient, which is proportional to the spontaneous Raman scattering cross section, and inversely proportional to the linewidth of the corresponding Raman line. Commonly, SRS is observed in two forms. The first, Raman generation, describes the Stokes-beam growth in the material from spontaneously scattered Raman-shifted radiation. The second, Raman amplification, is obtained when the energy from an intense pump beam is transferred to a weaker signal beam, which can be copropagating or counterpropagating [8,9,10,11,12,13,14,15,16].

Integrated nonlinear optics devices have been investigated since the 1970s. The attractive features of nonlinear waveguides are: 1) light intensity can be confined within an area comparable to the wavelength of light; 2) the diffractionless propagation in one or two dimensions results in interaction lengths over a distance (about a few cm) much longer than the one obtained with a bulk material. Since the nonlinear interaction efficiency depends nonlinearly upon the interacting beam intensities (power/area), and it is also proportional (either linearly or quadratically) to the interaction distance, the waveguide geometries offer the best prospects for optimizing the efficiency of nonlinear devices. Various types of all-optical functionalities, which can be significant for all-optical telecommunication networks, can be implemented by nonlinear integrated optical devices, based on different kinds of optical nonlinearities. They include signal regeneration, wavelength conversion, optical switching, routing optical demultiplexing and optical delay/buffering. At present the issue to be overcome is the materials’ performance. In the last decades, there has been a significant progress in this area with semiconductors and organic materials. However, there is still a key trade-off among the propagation losses, the nonlinearity and the nonlinearity response time, which does not allow the realization of high-speed, nonlinear devices performing at low optical power. The propagation losses limit the effective device length, and combined with nonlinearity, define the required device power. Due to the diffraction limit, a further limitation is that waveguide components do not allow confining light to the microscale or nanoscale dimension [17,18].

Microphotonics explore light behavior on the microscale and its interaction with micro-objects. The aim of microphotonics development is to go beyond the limit of photonics, offering a reliable platform for dense integration. The key challenges for microphotonics are a reduction in the size of integrated optical devices, and an improvement of performances with respect to nonlinear waveguide devices. During the last decades, the fast growth of micro-scales fabrication techniques has enabled the successful demonstration of various types of microphotonics devices, for example ring resonators and photonic crystals (PhCs) [19]. In a microphotonics device, photons are trapped in small volumes close to the diffraction limit for sufficiently long times [20], so that these photons strongly interact with the host material, creating enhanced nonlinear [21], quantum [22] and optomechanical [23] effects. We note that in this microphotonics device, although the physical phenomena observed are similar to the ones reported in a resonator etalon, the performance of micro structures involved is boosted by orders of magnitude. As a consequence, many physical phenomena have been observed with high compactness and integration, such as the Purcell effect [24], strong coupling between quantum dot, and cavity modes [25]. In nonlinear optical applications, microphotonics devices exhibit two interesting and useful aspects: the micrometers dimension and the increasing of the local field, combining a small modal volume with high optical quality-factors (Q) [26,27,28]. As an important consequence, a significant reduction of the power threshold of nonlinear optical effects is obtained.

Nanophotonics is a fascinating field, investigating the light behavior on the nanometer scale and its interaction with nanometer-scale objects [29,30]. We will have a big demand in the near future for devices, which should allow us to control light with light in a very thin nanoscale layer, or in a single nanoparticle of nonlinear material. In principle, in order to control a signal light in a nonlinear optical device, the intensity or phase of light has to be changed by a control signal, thus changing the optical characteristics of the medium. Of course, the stronger the nonlinearity of the material, the shorter the required interaction length *L*. We note that in nanoscale devices, the nonlinear effects cannot be enhanced using photon confinement effects, thus they only depend on the nonlinearity of the medium itself [31]. Therefore, a development of nanostructured materials with large nonlinearities, and satisfying also various technological and economical requirements [32], is mandatory. This is both an applicative issue, for an efficient device realization and design, and a fundamental issue, since the interplay between light and nanostructures is not yet understood.

Local enhancement and strong resonance of electromagnetic (EM) radiation in metallic nanoparticles and films continues to attract significant attention [33]. In these structures, a collective motion of conduction electrons (the surface plasmon polariton) becomes resonantly excited by visible light.

Bright, visible light emission in “bulk-sized” silicon coupled with plasmon nanocavities from non-thermalized carrier recombination was demonstrated by Cho, et al. [34]. However, although plasmonics allows a significant size reduction of optical components, the main drawback are still optical losses. On the other hand, owing to the big interest for the monolithic integration of photonic technology and semiconductor electronics, the EM radiation enhancement obtained from semiconducting and insulating materials is considerable [35].

In this review, the impressive progress, concerning an integrated laser source based on SRS and obtained in the last two decades, is described. In Section 2, for the sake of completeness, an essential theoretical background about SRS and a short introduction about the Raman laser in bulk are reported. In Section 3, the breakthrough of the first silicon laser is described. The possibility to obtain micrometer dimensions and the reduction of the power threshold to the microwatt pushed towards microphotonics structures. So, in Section 4 the most successful Raman lasers in microcavities and photonic crystals are reported. Finally, in Section 5, we discuss about SRS in nanostructures. We note that there are significant implications from both a fundamental and applicative point of view. From the fundamental one, a number of investigations, both experimental and theoretical, have been proposed in literature, but the “question is still open”, while from the applicative one, in order to realize micro-/nano-sources with improved performance, some encouraging perspectives have been pointed out [36,37,38,39]. Finally, recently proposed devices, combining silicon nanocrystals and microstructures, are reported, too.

## 2. Theoretical Background of SRS and Introduction to Raman Laser

**Figure 1:**
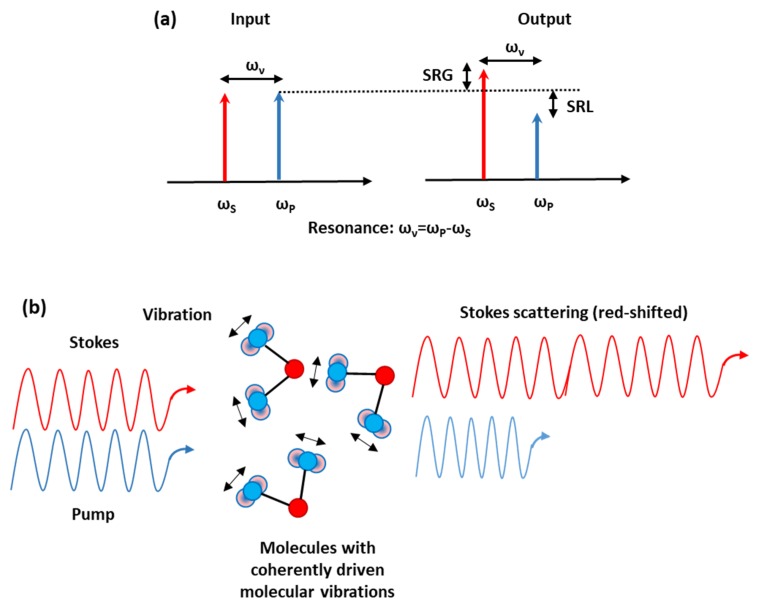
(**a**) SRS modalities: SRG, stimulated Raman gain; SRL, stimulated Raman loss; (**b**) inelastic scattering of probe photons obtained from vibrationally-excited molecules interfering coherently.

The SRS effect happens when a transfer of energy from a high power pump beam to a probe or Stokes beam (copropagating or counterpropagating) takes place. Precisely, this energy exchange occurs only if the frequency difference between the pump (at frequency *ω_P_*) and the Stokes laser beams (at frequency *ω_S_*) matches a molecular vibrational frequency of the considered material (i.e., *ω_S_* = *ω_P_* − *ω_υ_*, where *ħω_υ_* corresponds to a vibrational energy); the SRS effect leads to a gain of the Stokes beam power (stimulated Raman gain, SRG) and to a loss of the pump beam power (stimulated Raman loss, SRL) [8,9,10,11,12,13,14,15,16], as showed in Figure 1a.

SRS belongs to a class of nonlinear, optical processes called quasi-resonant. Although none of the two fields is in resonance with the vibrations in the lattice of the medium (optical phonons), their difference equals the transition frequency. The origin of SRS can be understood in terms of a two-step process: Frst, the pump produces frequency sidebands (Stokes and anti-Stokes) due to molecular vibrations; second, the Stokes wave, beating with the pump wave, produces a modulation of the total intensity, which coherently excites the molecular vibrations. These two steps strengthen each other, so the pump effect induces to a stronger Stokes wave, which in turn brings to stronger molecular vibrations. Because of its coherent nature, in SRS, the molecular bonds oscillate with a constant phase relation (see Figure 1b), interfering constructively inside the focus area of the laser beam, and therefore the SRS signal is orders of magnitude stronger than spontaneous Raman scattering. In SRS, the detection of a macromolecule with thousands of identical vibrational modes that interfere coherently is feasible. (see Figure 1b).

In SRS, high-order Raman sidebands can be produced by a field propagating through a medium optically polarizable. Many Stokes frequencies can be generated at the output when the pump power exceeds the threshold value, thus the Stokes frequencies at *ω_P_* − *ω_ν_*, *ω_P_* − 2ω_ν_ and those of anti-Stokes frequencies at *ω_P_ + ω_ν_*, *ω_P_ + 2ω_ν_* can be observed. If the intensity of the first-Stokes wave is high enough, it can generate a ‘‘second-Stokes’’ beam. By iterating this process, a higher Stokes order can be generated, leading to the so-called ‘‘cascaded’’ SRS. The intensity of the *i*^th^ Stokes order thus depends on the conversion rate from the *(i − 1)*^th^ Stokes order (proportional to *g·I_i_·I_i-1_*) and the loss rate to the *(i + 1)*^th^ order (proportional to *g·I_i_·I_i+1_*), where *g* is the Raman gain of material [8,9,10,11,12,13,14,15,16]. The generation of a coherent radiation in a wide interval of wavelengths, from the ultraviolet to the infrared can be obtained within a single Raman laser system, by combining conventional diode-pumped, solid-state lasers and different Stokes orders of SRS provided by a cascading effect [40,41,42,43].

In bulk semiconductors, lasing by SRS was first discovered in GaP [44]. Raman lasers (RLs) are similar to ordinary lasers. A first analogy is that lasing in RLs occurs when the Raman-active gain medium is placed inside a cavity to achieve the laser threshold. A second analogy is that the threshold power in RLs is obtained when the Raman amplification during a round trip is as large as to compensate the cavity losses. However, there are also some important differences between RLs and traditional lasers. A first one is that an amplifier medium based on Raman gain is used rather than on stimulated emission from excited atoms or ions. A second difference is that the required wavelength for pumping the Raman laser does not depend on the electronic structure of the medium, so it can be chosen to minimize absorption.

A Raman-laser-based approach takes advantage of the huge developments achieved in the engineering of diode-pumped, solid-state lasers, and the existing number of excellent Raman laser crystals. Moreover, this approach offers many attractive features. The main advantage is that in principle, any Raman laser wavelength can be obtained by a proper choice of the pump wavelength, when both wavelengths are within the transparency region of the material, and an adequately high nonlinearity and/or optical intensity are provided. The tunable output wavelength is a very important feature of crystalline Raman laser sources, since it can lead to several applications for Raman-laser-based sources working in the UV range, such as in remote sensing or the detection of explosives and biological agents, biomedical, including drug discovery, mass spectrometry and fluorescence imaging. Regarding the visible range, applications are in areas such as ophthalmology, biomedicine, dermatology, display and remote sensing [43]. Moreover, the Raman-laser-based approach shows excellent prospective for miniaturization, high beam quality, relative easy design based on commercially available components (and hence low cost), possibility to tailor output properties, potential to scale to multi-watt powers, and possibility to implement both CW and pulsed mode.

Numerous design options for lasers employing SRS, which can be used to tailor Raman laser characteristics and performance, have been developed. The basic configuration is the Raman generator, where a pump beam is focused (or telescoped) into a Raman crystal in order to generate a Stokes beam. This configuration can be used when the Raman gain is very large, the laser intensity can increase from noise to a significant signal without feedback. Typically used with short pulse (picosecond (ps)) lasers, this process is capable of generating a large number of Stokes and anti-Stokes lines, and yields efficiencies as high as 95%.

**Figure 2:**
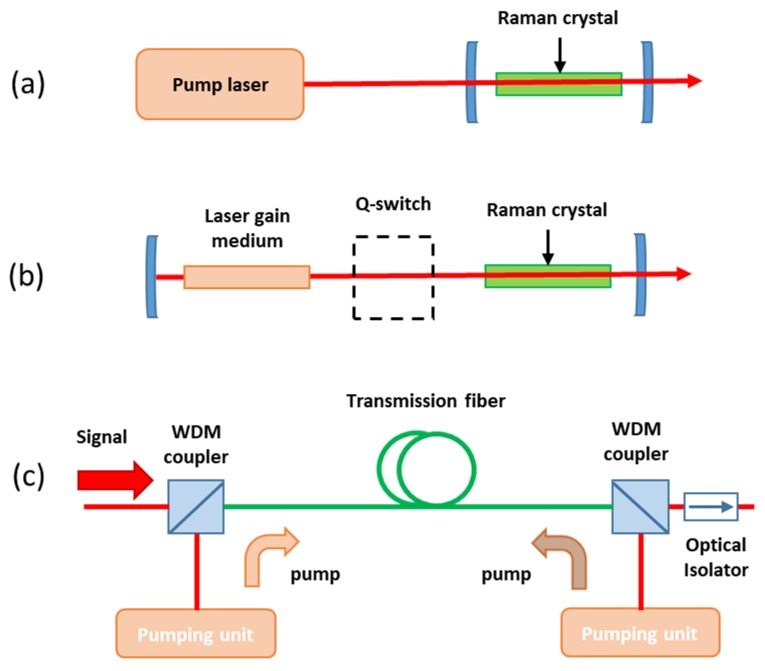
Typical configurations of the Raman laser: (**a**) external-resonator Raman laser; (**b**) intracavity Raman laser; and (**c**) fiber Raman amplifiers.

For pump pulses longer than the transit time through the active medium, the Raman crystal is placed inside a cavity resonating the Stokes field. Basically, there are two main configurations to implement RL: (i) external-resonator RL, where the Raman crystal is placed inside a cavity, resonating the Stokes beam (Figure 2a); (ii) the intracavity RL (Figure 2b), where both a Raman medium and the laser medium are combined inside a single cavity, so the fundamental and Stokes fields are both resonating inside the cavity [41]. Incorporating the Raman material inside the laser cavity, the high intracavity powers can be used to allow the conversion of low power lasers. Moreover, fiber optics amplification has been obtained by means of the Raman effect [45]. In this case, being that optical fiber used as the Raman gain medium, both pump and Stokes beams are launched into it. (Figure 2c). However, considering the currently-available communication range of about 50 THz, the existing silica fiber amplifiers are limited by their narrow usable bandwidth for Raman amplification (5 THz, approx. 150 cm^−1^).

Cascading is an interesting approach for the multiple-wavelength and wavelength-selectable Raman lasers. In order to select the output wavelength from a single device, the cascading effect, in combination with second-harmonic generation and or sum-frequency generation (SHG/SFG), provides a high degree of flexibility. SRS and SFG/SHG can be carried out either intracavity or extracavity, therefore a range of configurations can be developed to provide the selection of wavelengths.

**Figure 3:**
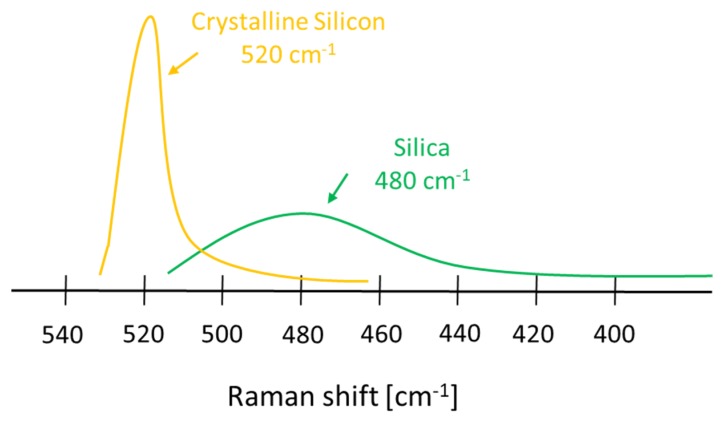
Comparison of Raman bandwidth and the efficiency of silicon and silica.

As general rule, all laser gain bulk materials have a tradeoff between gain and bandwidth, such that linewidth may be increased to the detriment of peak gain. This trade-off is a significant limitation towards micro-/nano-sources realizations with large emission spectra, for which a nonlinear material with wide, flat and high Raman gain in the range of interest [46,47] should be individuated. We consider, for example, glasses and silicon, which have been two leading materials for application in fiber and integrated photonics, respectively. Silicon has a high Raman gain and small bandwidth, whereas silica has a large bandwidth, but a small Raman gain; in Figure 3 we reported as an example the Raman spectra of the two extreme cases of crystalline silicon (c-Si) and silica. In order to answer the telecommunications demands, the investigation of new materials with both large Raman gain coefficients and spectral bandwidth is required. [48,49,50,51,52,53,54,55,56,57,58].

## 3. The First Silicon Laser

Silicon (Si) is considered the principal material for the microelectronics industry, but this is not yet true in photonics, due to its indirect bandgap. However, due to its wide bandwidth, high speed and low power dissipation, optical interconnect is emerging as an encouraging approach for on- and off-chip communications; therefore, it is viewed as the natural replacement of the electronic component in data transmission. For these reasons, silicon photonics is considered a technology on which to invest, because silicon-based optical components could be realized, taking advantage of existing complementary metal–oxide–semiconductor (CMOS) silicon fabrication techniques. The final goal is to integrate both electronic and optical functions on the same chip. With this aim, a silicon-based light source [59], a silicon waveguide, a silicon optical modulator [60], and a silicon-based photodetector [61] should be developed. While passive silicon devices were proposed and developed in the 1990s [62], the design of active devices seem to be much more challenging, due to silicon hostile physical properties, such as an indirect bandgap that does not allow efficient optical transitions, and the almost lack of Pockel’s effect caused by a centrosymmetric crystal structure [63]. Therefore, crystalline silicon shows relatively poor linear optical properties, thus light emission is precluded. In the last years, several approaches were proposed to realize Si-based light sources and amplifiers; they can be classified in three main categories:

- Using spatial confinement of the electron in order to overcome the indirect band structure;
- Using optically active dopants obtained by the introduction of rare earth impurities;
- Take advantage of Raman scattering in order to achieve optical gain.

Taking advantage by nonlinear effects shown in silicon, nonlinear silicon photonics [64,65] has attracted a lot of interest to obtain light amplification, light generation and wavelength conversion [66,67,68,69,70]. Being high quality silicon-on-insulator (SOI) wafers currently commercially available, the silicon waveguide in SOI can be easily produced by a standard CMOS fabrication technique.

**Figure 4:**
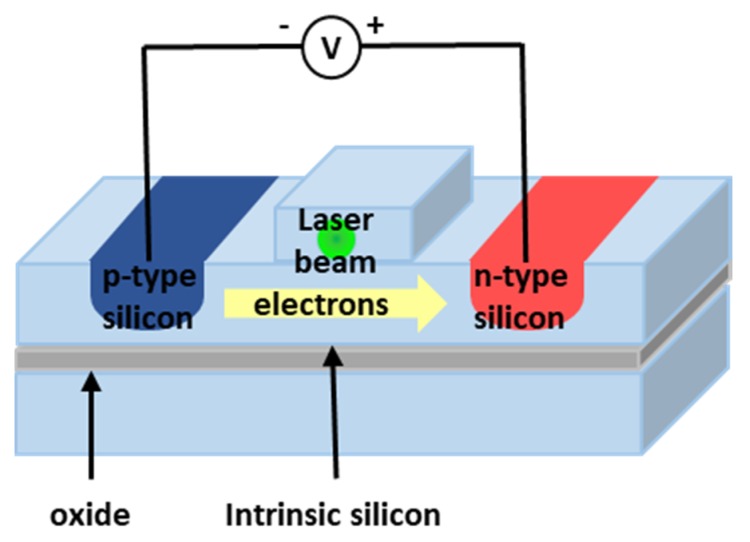
Typical configurations of silicon-on-insulator (SOI) waveguide Raman laser.

In SOI waveguides, the optical field can be confined to an area that is about 100 times smaller than the modal area of a standard single-mode, optical fiber, additionally due to the difference between crystalline and amorphous materials [71], the Raman gain cross-section in c-Si is five orders of magnitude larger than in silica. Thus, the silicon waveguide could be used to fabricate integrable and efficient amplifiers. The waveguide approach is schematically reported in Figure 4. We note that this Raman silicon laser was limited to centimeter-sized cavities with thresholds higher than 20 milliwatts [72,73].

The Raman approach allows one to avoid rare earth doping, such as erbium, making it fully compatible with silicon microelectronics manufacturing. On the other hand, the main limitation of the Raman approach is that electrical excitation is needed, requiring an off-chip pump. Another limitation is related to the two-photon-absorption (TPA), which gives rise to pump depletion and the generation of free carriers. TPA, through the free carrier plasma effect, leads to a broadband absorption spectrum. While the pump depletion is negligible since the TPA coefficient, β, is rather small (∼0.5 cm/GW) [74], absorption by TPA-generated free carriers is a broadband competing process with respect to the Raman gain, becoming a limiting factor in all-optical switching in III-V semiconductor waveguides, which is added to the linear optical scattering loss due to the waveguide sidewall roughness [75]. Moreover, because of the nonlinear optical loss associated with TPA-induced free carrier absorption (FCA) [76,77,78] in silicon waveguides, both the pump and signal beam decrease, making SRS not possible.

In order to overcome free carrier absorption, the first example of a silicon amplifier was based on a pulsed pump beam with a pulse duration shorter than the lifetime of the free carriers. The realized waveguide was 3 cm long, and its output signal was looped back into the entry by using an 8 m long single mode optical fiber; thus, an optical cavity was formed to achieve lasing [79].

The first observation of net optical gain in a low loss silicon waveguide in SOI, based on stimulated Raman scattering with a pulsed pump laser at 1.545 μm, was reported from the Intel Corporation [79]. A silicon rib waveguide was designed and fabricated on the (100) surface of a SOI substrate using standard photolithographic patterning and reactive ion etching techniques, and its effective core area was evaluated to be ∼1.5 μm^2^. With the aim to increase the interaction length, but at the same time have a micrometer-sized device, the waveguide was realized in an S-shaped curve with a bend radius of 400 μm and a total length of 4.8 cm. The measured linear loss was of 0.22 ± 0.05 dB/cm for both TE and TM modes; nevertheless, due to the strong pump beam, additional nonlinear optical loss in the silicon waveguide was observed and ascribed to TPA, since the two-photon energy of the pump beam is greater than the energy band gap of silicon. By using pulsed excitation, a decrease of the TPA-induced FCA allows obtain a Raman gain greater than the nonlinear optical loss. The reported net gain for such structure was of 2 dB, with a peak pump power of 470 mW and a pulse width of ~17 ns [80].

In 2005, after one year from the previous result, the same group demonstrated the first all-silicon-pulsed Raman laser [72]. The laser cavity was made by placing a mirror on one side of the 4.8 cm long, S-shaped silicon rib waveguide. The mirror was broadband, and had a high reflectivity (∼90%) for both the pump and the Stokes wavelengths, of 1.536 μm and 1.67 μm, respectively. The other side of the waveguide was used to couple the pulsed pump beam into the cavity, and had a reflectivity of ∼30% for both pump and Raman wavelengths. This solution allows us to weakly overcome the narrow-band (105 GHz) of stimulated Raman gain in Si that makes it inappropriate for use in wavelength division multiplexing (WDM) applications, unless expensive multi-pump schemes should be implemented. Indeed, by changing the cavity length it is possible to obtain different wavelengths out of a single pump wavelength within a very narrow interval. To overcome the TPA-induced FCA associated with Raman scattering, and thus to definitely reduce the accumulated carrier density inside the silicon waveguide, the authors proposed the use of a reversely biased p-i-n diode. They realized a p-i-n diode structure along the rib waveguide, and demonstrated that when a reverse bias is applied to it, the TPA-generated electron–hole pairs can be carried away from the silicon waveguide between the p- and n-doped regions by the applied electric field. Therefore, by changing the reverse bias voltage, the carrier transit time or effective carrier lifetime can be modified. The laser output power was measured at the uncoated side of the silicon waveguide cavity with a reverse bias of 25 V; in this condition, the obtained laser threshold was at ∼0.4 mW, and the slope efficiency (single side output) was 9.4%.

Afterwards, always the same group reported a continuous-wave (CW), all-silicon Raman laser [81]. The previous presented optical cavity was suitably modified to obtain CW lasing. In detail, both sides of the low-loss, S-shaped silicon waveguide were coated with multilayer dielectric films to reduce cavity loss. The front facet coating was a dichroic with a reflectivity of ∼71% for the Stokes wavelength (1686 nm) and ∼24% for the pump wavelength (1550 nm). Regarding the back side, it was designed to have a broadband high-reflectivity coating (∼90%) for both pump and Raman wavelengths. With these values of reflectivity, a low finesse cavity at the pump wavelength was obtained, allowing us to get the pump power cavity enhancement effect to lower the lasing threshold. If the pump laser matches the resonance of the cavity, the power inside the waveguide cavity is improved, leading to a decrease of the power threshold. Here an electric field generated by a reverse biased p-i-n diode drives away the free carrier generated by TPA in the waveguide channel. These free carriers are then collected by two electrodes, leading to a stable CW laser emission. The reported lasing thresholds were of ∼180 mW with a 25-V bias and ∼280 mW with a 5-V bias. This difference in the threshold depending on the bias voltage is due to lower nonlinear loss linked to the reduction of the effective carrier lifetime, as expected. The slope efficiency (single side output) above threshold was ∼4.3%, with a reverse bias of 25 V and 2% with a 5-V reverse bias. The p-i-n diode has been used also with a forward bias to inject free carriers in the silicon waveguide to absorb light and switch off the laser emission [82].

Tyszka−Zawadzka et al. have presented a semi-analytical model of Raman generation in the SOI rib waveguide with a DBR/F−P resonator [83]. The authors have proposed an approximate, semi-analytical expression relating the Raman output power to the pump power and system parameters, taking into account the Raman amplification, as well as the linearly-distributed losses in the laser cavity, the linear effects related to the FCA, and the nonlinear absorption associated with the TPA-induced free carrier absorption. A slight influence of TPA on the threshold pump power and output power of the SOI laser was highlighted by their numerical results obtained for the threshold laser operation and the above threshold laser operation. Another sophisticated and integrated multiphysics algorithm procedure was developed to accurately predict wave evolution and power transfer (pump, Stokes) propagating in the SOI microcavity resonator by taking into account the thermal and stress influences on the SRS and on all other linear and nonlinear physical effects involved in the Raman lasing mechanism [84].

## 4. Raman Laser in Microphotonics

In microstructures, the SRS enhancement, which is attributed to the photon confinement effect, can be evaluated by an equivalent gain (g_micro_), given by g_micro_ = f × g_bulk_, where g_bulk_ is the gain of bulk material making up the microstructures, and f is the optical field enhancement related to the presence of microstructures. We note that the bandwidth of the Raman gain does not change, and therefore, using microstructures, the fundamental trade-off between the gain and bandwidth of bulk materials cannot be overcome.

### 4.1. Raman Laser in Microcavities

Regarding SRS, it was observed that, within a droplet microcavity, the Raman gain improvement is inversely proportional both to the linewidth of the Raman process and to the square of the radius of the spherical cavity [85].

Spillane et al. studied SRS in spherical droplets and silica microspheres, with diameters of the order of tens of micrometers, and optically coupled by the use of a tapered optical fiber [35]. The authors measured the threshold power, whereas the coupling air gap between the taper and the microsphere was changed, and they found that in such way a nonlinear Raman source with a pump threshold approximately 1000 times lower than reported before, and a pump-signal conversion higher than 35%, can be obtained [46,86].

A glycerol–water droplet on a superhydrophobic surface coated with silica nanoparticles was used, as stationary microdroplets to achieve Raman lasing, by Sennaroglu, et al. [87]. By exciting a 12.4-μm-diameter droplet with a 532 nm Nd:YAG laser, both cavity-enhanced Raman scattering and Raman lasing centered at 632.3 nm were observed. Moreover, a typical on/off behavior of Raman lasing in microdroplets was reported and ascribed to the thermally-induced fluctuations during lasing. An improvement of the lasing effect can be obtained by increasing the rate of convective cooling, making this system useful as a light source for short-haul communications systems [87].

**Figure 5:**
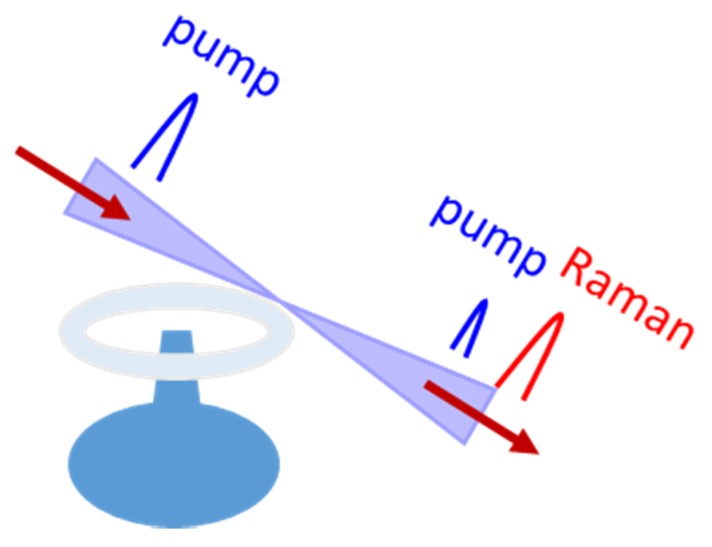
Schematic example of a taper-toroid coupling system.

A complete study of Raman oscillation in fiber-taper-coupled microspheres and microtoroids on-a-chip, from both theoretical and experimental points of view, was carried out by Kippenberg et al. [88]. With respect to Raman oscillation in microspheres, microtoroids show both power efficiency and spectral benefits, as well as having advantages with respect to their chip-based fabrication. Additionally, in the microtoroid-based device, single-mode oscillations are allowed due to a high reduction in the complexity of the mode spectrum, and for comparable Q-factors, lower threshold pump powers can be achieved due to a controllable and reduced mode volume. In particular, the threshold power reduction will depend upon the “aspect ratio” of the toroid given by *D*/*d*, where *D* = 50 μm is the outer diameter, and *d* is the minor one. The case of *d* = 50 μm corresponds to a sphere. A schematic example of the microtoroid-based device is reported in Figure 5.

In 2011, the influence on the Raman signal in a single microsphere of several parameters, such as the pump wavelength, size and refractive index of the microsphere, and the numerical aperture of the microscopic objective lens, was analyzed [89]. Results showed that, due to the increased field of the photonic nanojet emerging from the single microsphere, an enhancement ratio of silicon wafer and cadmium ditelluride Raman peaks, approximately of two orders of magnitude, can be obtained by suitable selection of the experimental parameters [89].

Taking advantage of the third-order nonlinearity χ(3) in a microresonator, the four-wave mixing (FWM) process can be used to generate a broadband frequency comb using parametric oscillation. Using a silicon microresonator, the first low-noise coherent mid-IR frequency comb source was obtained [90,91]. As a result of the interaction between the stimulated Raman effect and FWM, phase locking of the generated comb can be observed, leading to strong comb lines separated by the Raman shift in silicon [90,91].

Alternatively, diamond was suggested as a reasonable material for compact, on-chip Raman lasers over a wide spectrum [92]. A CW low-threshold Raman laser, based on waveguide-integrated diamond racetrack microresonators embedded in silica on a silicon chip, was demonstrated in [93]. Pumping at telecom wavelengths, a tunable Stokes output over a ∼100 nm bandwidth around 2 μm, with output power > 250 μW, was reported.

In 2017, a tunable Raman laser in the hollow bottle-like microresonator (BLMR) with a high-Q factor of 2.2 × 10^8^ was demonstrated [94]. Continuous output frequency tuning was obtained by controlling the pump laser frequency or power through the thermal effect; the tuning range was of 1.2 nm, corresponding to a frequency range of 132 GHz, with a minimum tuning step of about 85 MHz. Additionally, a large range frequency tuning of the Raman laser, with the tuning range of 132 GHz and a resolution of about 85 MHz, was also demonstrated by mechanically stretching the resonator. These approaches could open the way to future optical applications of WGM microresonators [95,96].

### 4.2. Raman Laser in Photonics Crystals

Photonic crystals were introduced for the first time in 2005 to enhance stimulated Raman amplification and lasing in monolithic silicon [97]. Indeed, due to their increased light–matter interactions, these structures seem to be good candidates to permit the realization of ultracompact silicon Raman light amplifiers and lasers.

An L5 photonic bandgap nanocavity in two-dimensional photonic-crystal slabs was realized, and the power threshold estimated was about 130 μW, considering parameters *g_s_*=70 cm/GW, ξ = 1 (modal overlap), *λ_s,p_* = 1550 nm, *Q_pump_* = 1550 and *Q_Stokes_* = 4.2 × 10^4^ (*Q_pump,Stokes_* are the cavity quality factors). The threshold can be further reduced to tens of microwatts by designing ultrasmall, photonic, bandgap nanocavities with higher-Q factors [98,99]. Indeed, a drastic enhancement of the Raman gain beyond that predicted theoretically can be achieved by designing nanocavities considering that the strength of light–matter interactions is proportional to the ratio of the quality factor to the cavity volume [99].

Regarding the photonic crystal waveguide (PCWG), a number of experiments have been performed in the so-called W1 waveguides obtained by removing a single line of holes in the ΓK direction of an otherwise perfect photonic crystal [100,101]. A theoretical study of the SRS enhancement in a slow-light silicon PCWG through a four wave-mixing formalism from the computed modes of the line-defect waveguide was carried out by McMillan et al. [100]. In detail, by comparing the group velocities of Stokes and pump signals, an enhancement of the stimulated Raman gain up to approximately 10^4^ times compared to bulk Si was demonstrated. Afterwards, the same group demonstrated an enhancement of the spontaneous Raman scattering coefficient higher than six times, due to the slow light in silicon PCWG [101].

However, these structures are resonant only at the Stokes frequency, thus SRS occurs under pulsed excitation, while in a continuous regime the Raman-scattering efficiency is not high enough with respect to the efficiency of other competitive nonlinear effects, such as TPA and FCA.

In order to increase the spontaneous and stimulated Raman-scattering efficiency, structures resonating at both the pump and Stokes wavelengths should be implemented. In 2010, Checoury et al. investigated spontaneous and stimulated Raman scattering in very narrow W0.66 PCWG (width of the W0.66 = 2/3 of the W1 standard width) [102]. The realized waveguides were oriented along the (100) crystallographic direction of silicon, and allow both a 60% decrease in the Raman volume with respect to W1 waveguides oriented along the (110) direction, and a decrease in both the pump and Stokes modes group velocities. SRS was observed at room temperature for a continuous incident power as low as 20 mW, in good agreement with the simulations performed considering FCA negligible.

A Raman laser was made in 2013 by a photonic crystal high-quality-factor nanocavity with a dimension less than 10 μm [103]. The photonic crystal had a triangular lattice structure composed by circular air holes in a suspended silicon membrane. The nanocavity was realized by a linedefect waveguide that shows two types of propagation modes within the photonic bandgap, an odd-waveguide mode and an even-waveguide mode, allowing us to confine pump light and Stokes–Raman-scattered light, respectively. This structure gives rise to a CW Raman silicon laser with a low lasing threshold of 1 μW. Moreover, this Raman laser does not require p–i–n diodes, because the nanocavity produces net Raman gain in the low-excitation range before TPA-induced FCA becomes dominant, allowing this low lasing threshold [103].

## 5. Raman Laser in Nanophotonics

During the past few decades, a significant number of nanomaterials have shown to have notable optical properties, motivating the fabrication and design of nanoscale photonic devices [104]. These nanoscale devices are important in order to obtain a smooth integration with electronic devices, for example, transistors in sub-100 nm length scales. In this context, one of the most promising materials for light emission applications in microphotonics is based on silicon nanocrystals (Si-nc). The main idea is to induce quantum confinement effects by limiting carriers into very small silicon nanoclusters (1–4 nm in size), leading to change in the physical properties of bulk silicon. In detail, the reduction of dimensionality affects not only the linear optical properties, such as emission efficiency and band gap, but also the nonlinear optical properties, which are usually enhanced [93,105]. Recently, third-order, nonlinear, optical properties of Si-nc have been investigated, and a significant variation of the nonlinear refractive-index (n_2_) values has been shown up to two orders of magnitude in SiO_2_ films containing Si-nc and/or Si nanoclusters, with respect to crystalline silicon. Therefore, the comparison between experimental and theoretical results is still an issue [106]. From a theoretical point of view, it has been demonstrated [107], that in order to explain the origin of large values of third-order, nonlinear, optical properties, which are affected by their structural parameters (crystallinity, density, size and distribution), both the defect states and the quantized electronic states should be taken into account.

Concerning spontaneous Raman scattering, a strong enhancement (∼10^3^) obtained from individual silicon nanowires and nanocones, as compared with bulk Si, was reported by Cao et al. [108]. The increase in Raman-scattering intensity with decreasing diameter was explained by structural resonances in the local field.

Concerning SRS in silicon nanocrystals (Si-nc), a giant Raman gain was measured at the wavelength of interest for telecommunications. The film of silicon nanocrystals (Si-nc), embedded in a silica matrix and obtained by the molecular beam deposition method, had an increasing Si concentration along the longer dimension. An impressive enhancement of the Raman gain in Si-nc up to four orders of magnitude, compared with bulk silicon, as a function of Si concentration, was observed [56]. We note that the volume of Si-nc does not exceed 10% of the volume of the sample, which makes the difference between the gain properties of Si-nc and bulk silicon more significant. From the applicative point of view, these experimental results open the way towards Raman–laser-based nanostructured material. Since the SRS effect in Si-nc about 10^4^ times larger with respect to silicon, a Raman laser with typical dimensions of a few micrometers could be obtained.

Therefore, all the advantages of combining optical and electronic functions on a single chip [56] could be experienced.

Although a general theory on the relation between nanostructuring and Raman gain is not established, we expect that when the particle dimensions are of a few nanometers, the phonon confinement effect plays a significant role; therefore, SRS enhancement can be attributed to the quantum confinement effect. Therefore, the gain of nanomaterials should be different from bulk, and related to the intrinsic properties of materials, and the essential trade-off between gain and bandwidth should be overcome, too. We guess that in Si-nanocrystals (Si-nc), the physical insight is similar to the one reported by Peng in [37]. When the mean free path of an electron is larger with respect to a phonon, the electron can collide with the phonon many times, and a strong phonon amplification can be obtained. From the point of view of energy transfer, first the energy of the laser field is absorbed by the electrons, and then it is transferred to the phonons by the electron–phonon interaction. If a resonance condition is obtained, for example, due to the interface levels, the movement of electrons is strongly confined, and due to the confinement effect, we expect that all light-generated electrons have to be involved in the electron–phonon interaction, resulting in significant amplification of phonons. Definitely, the efficiency of the electron–phonon interaction in a nanocrystal is much higher than in a bulk crystal [38].

In reference [109], Agarwal et al. reported a strong SRS and very high Raman gain in optical cavities made of Si nanowire of various diameters in the visible region [109]. The authors evaluated by electromagnetic calculations an enhancement of the Raman gain coefficient of Si nanowire by a factor greater than 10^6^ at 532 nm excitation with respect to the gain value at the 1.55 μm wavelength reported in literature [56], even though the losses are 10^8^ higher at 532 nm. They ascribed this behavior to the higher electric field intensity and lower mode volume of the electromagnetic modes inside the Si nanowire, with respect to bulk Si at the pump and the Stokes wavelength, as well as the spatial overlap of these modes inside the cavity. Moreover, they measured the SRS threshold as low as 30 kW/cm^2^. These results could allow the realization in the next future of a monolithically-integrable, nanoscale low-powered Si Raman laser.

Recently, Rukhlenko and Kalavally presented the first theoretical study of CW Raman amplification in silicon–nanocrystal waveguides with enhanced mode confinement [110]. In detail, they studied Raman amplification in silicon nanocrystal waveguides, and observed that it strongly depends on the composition and geometric parameters of the waveguide. Fixing the geometric parameters of the waveguide, the maximal Stokes intensity peaks can be obtained for a given optimal density of silicon nanocrystals. Moreover, the optimal length and peak Stokes intensity depends on the height and width of the waveguide’s cross-section and input conditions. However, the amplifier again requires a moderate power off-chip laser pump, and also the waveguide length is yet not small enough in view of dense integration.

Interestingly, slotted PCWGs performance are highly dependent on the width and refractive index of the slot. This dependence was studied and formalized by Datta’s group [111], by calculating the respective dispersion diagrams using the three-dimensional full-vector Plane Wave Expansion method. They also characterized the nonlinear performance of these slotted PCWGs by using a low-index, highly nonlinear material in the slot. In particular, a silicon nanocrystal-embedded PCWG was designed to enhance the SRS gain, and this enhancement shows a net gain of the order of 11 dB in a ~7 µm long slotted PCWG.

Thereafter, the same group, exploiting their theoretical study, as well as the giant Raman gain of silicon nanocrystal material [56], proposed an all-silicon, micron-scale, Raman amplifier based on SiNC∕SiO_2_ embedded in slotted PCWG [112]. In detail, the structure consisted in a suspended silicon slab, which is pierced by holes of air to form the photonic crystal. The holes were organized in a triangular lattice (lattice constant a = 0.426 µm), and their radius, the thickness of the slab and the width of the SiNC∕SiO_2_ slot, have been taken as 0.3a, 0.7a and 0.2a, respectively. A volumetric proportion of approximately 1:10 of Si to the SiO_2_ leads to a refractive index of 1.98 of the SiNC∕SiO_2_. In order to obtain both a low-cost optical pumping and the possibility of on-chip integration, a Light Emitting Diode (LED) with 6 mW pump power has been considered.

The authors demonstrated that an amplifier made with a 4 µm long slotted PCWG yields to an overall gain of ~3.22 dB at a bit rate of 400 Gbps, whereas with a 10 µm long slotted PCWG the gain is increased to ~7.93 dB at 200 Gbps. Finally, they suggest to exploit the strong electroluminescence from SiNC∕SiO_2_ in order to integrate the pump source on the same platform, slowing in the next future to realize the micron-scale silicon Raman laser without any external pump source.

In reference [113], Pradhan et al. reported the design of an integrable all-silicon Raman laser of a foot print of 7μm, based on a slotted photonic crystal nanocavity, which takes advantage the giant Raman gain coefficient of a silicon nanocrystal [56]. The device exhibits a lasing efficiency of 18% at a wavelength of 1552 nm, with an optical threshold power of the order of 0.5 μW. The effect of imperfections introduced during fabrication on the performance of the device was evaluated, too. Considering random variation in radii and in-plane positions of the air holes, the device performance was tolerant up to a 6% random variation of the structural parameters. In addition, the submicrowatt threshold of the device as a function of Q-factors and modal volume was evaluated, and it was demonstrated that it is tolerant for a significant deviation (over 30%) of these parameters from their optimized values.

**Figure 6:**
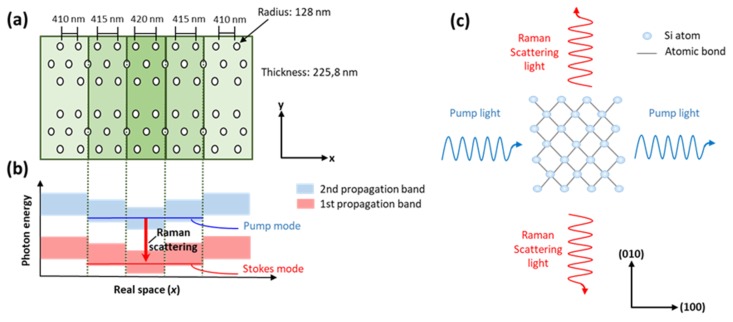
(**a**) Schematic of a heterosctructure nanocavity. (**b**) Band diagram of the nanocavity. (**c**) Schematic of the in-plane Raman scattering for the cavity’s x-direction being parallel to the (100) direction of crystalline Si.

More recently, a heterostructure nanocavity has been used to implement a Raman laser [114]. A line defect in a photonic crystal, made with a triangular lattice of circular air holes with a lattice constant a of 410 nm, defines the nanocavity, while the heterostructure is formed by increasing the lattice constant a by 5 and 10 nm in the x-direction in the areas closer to the center (Figure 6a, light and dark green areas, respectively) [115]. This change of the lattice parameter allows two propagation band frequencies that are the Stokes and pump modes, respectively (see Figure 6b), thus leading to the formation of two nanocavity modes with high Q. Figure 6c reports the in-plane Raman scattering in a microscopic model for the *x*-direction of the cavity chosen parallel to the [100] direction of Si. The polarization directions of the pump light and the Raman scattered light are orthogonal in the *x*–*y* plane in this geometry. The Si Raman laser was the realized on a modified (100) SOI wafer, in which the top Si layer has been changed by rotating the crystal orientation in the plane by 45° with respect to the crystal direction of the substrate. This device was characterized, and room temperature CW laser oscillation with a very impressive sub-microwatt threshold (0.53 μW), and a maximum energy efficiency of 5.6%, is reported [114].

## 6. Conclusions

In this review, the most significant results, concerning laser sources based on SRS obtained in the last two decades, are reviewed. After the description of first silicon laser, which was based on the nonlinear waveguide, we focus on microcavity and photonics crystals, which are able to enhance the nonlinear interaction between light and matter, allowing us to obtain promising integrated Raman active devices. Finally, some recent interesting investigations, combining the potentiality of silicon nanocrystals and microphotonics structures, have been reported, too.

Here, we try to highlight, not only the development of integrated Raman source, but also the transition between photonics devices realised in waveguide, and in microphotonics structures based on photon confinement effects. We try to elucidate the main difference related to their working principle and the advantages for their applications. From a theoretical point of view, we note that a general theory on the relation between nanostructuring and Raman gain is not completely established [116,117,118,119,120,121,122,123,124,125,126,127,128,129,130], while from an applicative point of view, reported results open new perspectives for the realization of more efficient Raman lasers with ultra-small sizes, which would increase the synergy between nanoelectronic and nanophotonic devices.

We note that while the transition between integrated waveguide and microphotonics structures has been facilitated by the development of micro-scales’ fabrication techniques, the one between micro and nano is still an issue. Right now, silicon nanocrystals are the more promising options. However, we note that SRS in nano is in its infancy, many investigations are not mature, and in some cases, only pioneering works or preliminary results on devices have been reported in the literature. Therefore the big challenge of the future is a reduction in the size of integrated optical devices towards nano dimensions, while maintaining a high level of performance.

We really hope that these encouraging perspectives can stimulate further theoretical and experimental works required to finally achieve the crucial milestone of a monolithically integrable, nanoscale, low-powered Si Raman laser, which could be integrated with other nanoscale electronic and optical components, leading to the development of next generation of nanosystems.
